# Impact of an integrated community case management programme on uptake of appropriate diarrhoea and pneumonia treatments in Uganda: A propensity score matching and equity analysis study

**DOI:** 10.1186/s12939-015-0202-y

**Published:** 2015-09-04

**Authors:** Agnes Nanyonjo, James Ssekitooleko, Helen Counihan, Frederick Makumbi, Göran Tomson, Karin Källander

**Affiliations:** Department of Public Health Sciences, Karolinska Institutet, Tomtebodavägen 18 A, Floor 4, Stockholm, SE-171 77 Sweden; Malaria Consortium Uganda Office, Plot 25, Upper East Naguru, P.O. Box 8045, Kampala, Uganda; Malaria Consortium, Development House, 56-64 Leonard Street, London, EC2A 4LT UK; Department of Epidemiology and Biostatistics, Makerere School of Public Health, Makerere University, New Mulago Hospital Complex, P.O. Box 7062, Kampala, Uganda; Medical Management Centre (MMC), Karolinska Institutet, Stockholm, Sweden

**Keywords:** Integrated community case management, Pneumonia, Diarrhoea, Equity, Treatment

## Abstract

**Introduction:**

Pneumonia and diarrhoea disproportionately affect children in resource-poor settings. Integrated community case management (iCCM) involves community health workers treating diarrhoea, pneumonia and malaria. Studies on impact of iCCM on appropriate treatment and its effects on equity in access to the same are limited. The objective of this study was to measure the impact of integrated community case management (iCCM) as the first point of care on uptake of appropriate treatment for children with a classification of pneumonia (cough and fast breathing) and/or diarrhoea and to measure the magnitude and distribution of socioeconomic status related inequality in use of iCCM.

**Methods:**

Following introduction of iCCM, data from cross-sectional household surveys were examined for socioeconomic inequalities in uptake of treatment and use of iCCM among children with a classification of pneumonia or diarrhoea using the Erreygers’ corrected concentration index (CCI). Propensity score matching methods were used to estimate the average treatment effects on the treated (ATT) for children treated under the iCCM programme with recommended antibiotics for pneumonia, and ORS plus or minus zinc for diarrhoea.

**Findings:**

Overall, more children treated under iCCM received appropriate antibiotics for pneumonia (ATT = 34.7 %, p < 0.001) and ORS for diarrhoea (ATT = 41.2 %, p < 0.001) compared to children not attending iCCM. No such increase was observed for children receiving ORS-zinc combination (ATT = -0.145, p < 0.05).

There were no obvious inequalities in the uptake of appropriate treatment for pneumonia among the poorest and least poor (CCI = -0.070; SE = 0.083). Receiving ORS for diarrhoea was more prevalent among the least poor groups (CCI = 0.199; SE = 0.118). The use of iCCM for pneumonia was more prevalent among the poorest groups (CCI = -0.099; SE = 0.073). The use of iCCM for diarrhoea was not significantly different among the poorest and least poor (CCI = -0.073; SE = 0.085).

**Conclusion:**

iCCM is a potentially equitable strategy that significantly increased the uptake of appropriate antibiotic treatment for pneumonia and ORS for diarrhoea, but not the uptake of zinc for diarrhoea. For maximum impact, interventions increasing zinc uptake should be considered when scaling up iCCM programmes.

## Introduction

Pneumonia and diarrhoea disproportionately affect children living in low-income countries. While there is evidence on effectiveness of community based delivery of treatment of pneumonia and diarrhoea [1–3], slow progress in coverage of these interventions has been noted over the last ten years [4–7]. Of critical importance is the extremely low coverage of oral rehydration salts (ORS) and zinc for the treatment of diarrhoea, despite their proven effectiveness [3, 8]. As a vehicle to increase coverage of lifesaving treatment for children suffering from these three child killers, a joint statement was produced by the World Health Organisation (WHO) and United Nations Children's Fund (UNICEF) in 2004 calling for integrated community case management (iCCM) of pneumonia and diarrhoea in addition to malaria in countries where these diseases are common [9]. Uganda was one of the first countries to respond to this call by formulating a national iCCM policy in 2010.

iCCM is now an increasingly common approach to tackling these diseases in Sub-Saharan Africa [1, 6, 9]. With respect to pneumonia, studies have previously estimated that community case management (CCM) has the potential to cause a 70 % reduction in mortality among children aged five years and below [10]. It is also postulated that CCM for pneumonia may lower drug resistance through improved social inclusion and rational drug use using the WHO approved classification and treatment algorithm [10, 11]. Despite these postulations, the introduction of antibiotics at the community level in African contexts has been criticised in some literature [12].

However, with the introduction of new health care interventions comes the risk of inequities in access to care for the poorest households [13, 14]. iCCM is inherently designed to improve access to health care for children in the poorest families but there is paucity of data on how iCCM has impacted on equity in access to appropriate treatment for diarrhoea and pneumonia. This study aimed to evaluate both equity in use of iCCM and its impact on uptake of appropriate treatments for diarrhoea and pneumonia when used as the first source of care.

## Methods

### Study setting

The study was conducted in nine predominantly rural districts in Midwestern Uganda where the iCCM programme had been implemented by Malaria Consortium in collaboration with the Ministry of Health since 2010. The districts had an estimated population of 2.2 million inhabitants and approximately 23.3 % were children below the age of five years. By 2012 approximately 6800 community health workers (CHWs) were trained to deliver iCCM in the area. The CHWs complemented the formal healthcare sector in an area where health markets for children range from informal care at home and in the private sector to formal care from often distant and poorly functional health facilities. More than half of children being taken for care outside the home in Uganda are known to go to a drug shop or private clinic [15].

In Uganda, CHWs working from home, locally known as village health team (VHT) members are an important and integral part of the healthcare system and represent the lowest health centre level offering promotive, preventive, and curative health services. In a typical village, there is a team of five to six CHWs offering a package of promotive and preventive health services, with two of them trained on iCCM for a duration of six days. Curative services offered under iCCM include provision of colour-coded artemether/lumefantrine combination to children aged 4–59 months confirmed to have uncomplicated malaria through a rapid diagnostic test, colour-coded amoxicillin to children aged 2–59 months with pneumonia classification based on cough and fast breathing, and zinc and ORS to children aged 2–59 months with uncomplicated diarrhoea. Children showing danger signs are referred to the nearest health facility, from which the CHWs receive supervision. In 2009 the recommended first-line antibiotic treatment for pneumonia was trimethoprim-sulfamethoxazole, while that for diarrhoea was ORS. Following a policy change in 2010, treatment guidelines changed to amoxicillin, and ORS-zinc combination for pneumonia and diarrhoea, respectively.

### Study design and participants

The outcome of interest in this study was receiving appropriate treatment for either pneumonia or diarrhoea and has been defined as a) a child receiving an appropriate antibiotic for pneumonia if he or she is reported to have had cough and fast breathing (fast breathing pneumonia) and b) a child receiving either ORS alone or a combination of ORS and zinc if he or she is reported to have had watery stools or abnormally frequent bowel motions. The exposure variable of interest in this study has been defined as obtaining treatment from a community health worker (CHW) delivering iCCM services.

In order to obtain data on the outcome and exposure variable of interest, the study draws from before (2009) and after (2012) population based household surveys. The surveys sought to a) evaluate effect of iCCM on the proportion of children with access to appropriate care for fever, diarrhoea and fast breathing pneumonia through assessment of caregivers health-seeking behaviour around the illnesses, and b) provide an indirect estimate of infant and under-five mortality before and after implementation of iCCM compared to control areas (data not presented in this paper but elsewhere [16]). With respect to health seeking behaviour, the surveys employed a facilitator-administered a questionnaire that asked about the symptoms experienced by eligible children, having sought for health care from outside the home, places from which health care was sought and any treatments obtained. The treatments obtained, when necessary were identified by the facilitators through examination of drug leftovers, prescription notes and with the use of ‘Drug cards’, which were a collection of pictures of common medicines created to assist caretakers in the identification of treatments provided for their children.

Study participants included primary caregivers of under-fives to whom the adapted questionnaire was administered. The sample size was therefore calculated based on a population-based, representative household interview survey for children under five, with a primary sample of 100 clusters for which household and birth history data were collected, and a smaller, nested sample of 40 clusters for the health-seeking behaviour component. Participant sampling was done with two-stage cluster sampling using the census database of the respective districts as a sampling frame, without restriction or exclusion. The primary sampling units were enumeration areas (village or cluster of household), which were selected with probability proportionate to size. First, the total sample needed for mortality estimation was drawn jointly with the Uganda Bureau of Statistics (UBOS) followed by a sub-set of clusters selected with equal probability, which were used for the health-seeking behaviour assessment. In the second stage of sampling, all households in the selected villages were listed and the number of households needed was selected by systematic sampling. For villages with more than 200 households, an equal size section approach was used whereby the village was divided into 2-4 sections with an approximately equal number of households, and one of the sections was selected using simple random sampling.

### Data analysis

**Fig. 1:**
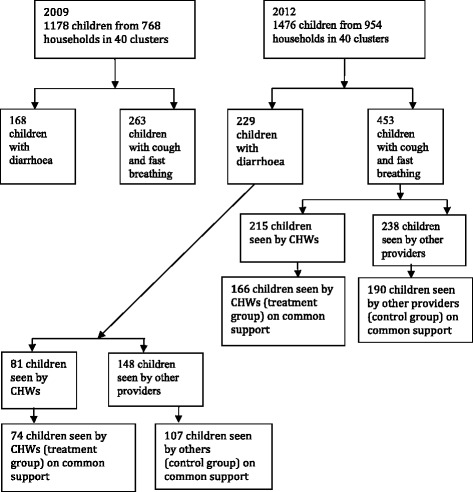
Flow chart showing the breakdown of sample sizes for secondary data analysis for uptake of pneumonia and diarrhoea treatments

The analysis in this paper is limited to the children aged 2–59 months whose primary caretakers were interviewed for health-seeking practices from the nested 40 clusters at baseline (2009) and after implementation of iCCM (2012). The data obtained in 2012 were used to assess equity in use of iCCM among children with a classification of pneumonia or diarrhoea and to evaluate the impact of iCCM on uptake of appropriate treatment for diarrhoea and pneumonia. Additionally, data obtained in 2009 were used for complementary descriptive analysis using before and after comparisons. The impact of iCCM on treatment uptake was evaluated using propensity score matching methods (PSM). Therefore, the sample size for impact evaluation included only matched samples in the region of common support described further in the analysis section [17, 18]. Figure 1 summarises how the sample size for this study was derived.

### Ethical considerations

Ethical approval for the study was obtained from the Uganda National Council of Science and Technology (HS 666). Written informed consent was obtained from all the study participants.

### Statistical analysis

The main objectives of the analysis were to measure the impact of iCCM on uptake of appropriate treatments for pneumonia and diarrhoea, and to measure the magnitude and distribution of socioeconomic inequality in use of iCCM. Uptake was defined as the proportion of children eligible for treatment who were reported to have received treatment. Children were eligible for pneumonia treatment if they were reported to have had cough and fast breathing while for diarrhoea it was children reported to have had watery stools or abnormally frequent bowel motions. Appropriate treatment for pneumonia comprised of child receiving any antibiotic that was recommended as a first line treatment according to Uganda’s treatment guidelines or iCCM protocol [19]. Similarly the national treatment guidelines and iCCM protocol recommend ORS and Zinc for diarrhoea and therefore appropriate treatment for diarrhoea comprised of ORS or a combination of ORS and zinc. For the uptake analysis, only the first source of health care from which the child’s caregiver sought for treatment outside the home was considered. Differences in the proportions of children treated appropriately at different time points and between the different groups were determined using Chi-square (*χ*^2^) tests. The study probed for inequalities in both the disease prevalence and reception of appropriate. During the inequality analysis, a socioeconomic status index was generated through principle components analysis of household assets and income generating activities that are recommended by the Uganda Bureau of Statistics [20, 21]. The variables used in the analysis included building materials for the household, water source, type of toilet, type of fuel used for cooking and occupation of the head of the household. The socioeconomic status index was then divided into wealth quintiles. The individual indicators that weighted heaviest in the analysis were house construction materials. The socioeconomic status of the population was ranked from poorest to least poor, and age-sex indirectly standardized concentration indices (CI) were used to measure socioeconomic related inequality in uptake of antibiotics for pneumonia and ORS and zinc for diarrhoea. The CI is a measure of socioeconomic related inequality, which takes on values between -1 and +1. In the absence of inequalities, the concentration index takes on the value of zero. Positive values of the CI imply that a situation disproportionately affects the richer groups and negative values imply that a situation affects the poorer groups more and the larger the value, the larger the degree of inequality. Since receiving appropriate treatment and using iCCM are both binary variables, the corrected version of the CI, known as Erreygers concentration index (CCI) recommended for bound outcomes, was computed together with its standard error [22, 23]. The index is denoted as:

1 $$ \mathrm{C}\mathrm{C}\mathrm{I}\ \left(\mathrm{y}\right) = 8\mathrm{c}\mathrm{o}\mathrm{v}\left(\mathrm{y}\mathrm{i},\mathrm{R}\mathrm{i}\right) $$

where Yi = binary outcome and Ri = Fractional rank is SES distribution. If inequality can be explained by linear regression of K (justifiable standardising factors) and J (non-justifiable inequality), this can be denoted as eq. (2)

2 $$ {\mathrm{y}}_{\mathrm{i}}={\upbeta}_0+{\displaystyle \sum_{\mathrm{k}=1}^{\mathrm{k}}}{\upbeta}_{\mathrm{k}}{\mathrm{x}}_{\mathrm{i}\mathrm{k}}+{\displaystyle \sum_{\mathrm{j}=1}^{\mathrm{J}}}{\upbeta}_{\mathrm{j}}{\mathrm{z}}_{\mathrm{i}\mathrm{j}}{\upvarepsilon}_{\mathrm{i}} $$

then equation (i) can be rewritten as equation (iii) to show socioeconomic related inequality in uptake as expressed as a weighted sum of inequalities in its determinants and a residual term.

3 $$ \mathrm{C}\mathrm{C}\mathrm{I}\left(\mathrm{y}\right)=4\left[{\displaystyle {\sum}_{\mathrm{k}=1}^{\mathrm{k}}}{\upbeta}_{\mathrm{k}\ }{\overline{\mathrm{x}}}_{\mathrm{k}}\mathrm{C}\mathrm{I}\left({\mathrm{x}}_{\mathrm{k}}\right) + {\displaystyle {\sum}_{\mathrm{j}=1}^{\mathrm{J}}}{\upbeta}_{\mathrm{j}}{\overline{\mathrm{z}}}_{\mathrm{j}}\mathrm{C}\mathrm{I}\left({\mathrm{z}}_{\mathrm{j}}\right)+{\mathrm{GC}}_{\upvarepsilon}\right] $$

With the means of k _x_ and Z_j_ denoted as ${\bar{\mathrm{x}}}_{\mathrm{k}}$ and ${\overline{\mathrm{z}}}_{\mathrm{j}}$ respectively, and their concentration indices denoted as CI (X_k_) and CI (Z_j_) respectively, where $\mathrm{G}{\mathrm{C}}_{\upvarepsilon}$ is a residual term, the contribution of each factor to the inequalities can thus be established. Unfair inequality can thus be measured by subtracting fair inequality from the corrected concentration index to give an index of horizontal inequity (I) [24].

$$ \mathrm{I}=\mathrm{C}\mathrm{C}\mathrm{I}\left(\mathrm{y}\right)\hbox{-} 4{\displaystyle {\sum}_{\mathrm{k}=1}^{\mathrm{k}}{\upbeta}_{\mathrm{k}\ }}{\overline{\mathrm{x}}}_{\mathrm{k}}\mathrm{C}\mathrm{I}\left({\mathrm{x}}_{\mathrm{k}}\right) $$

Due to the binary nature of the outcome variables, unjustifiable inequality was estimated through a linear model. Justifiable causes introduced in the model included: age and sex while non-justifiable causes included socioeconomic status, source of care, urban-location, education level and occupation of caregivers. Horizontal inequity was only calculated for the variable use of iCCM and not the variables relating to uptake of appropriate treatment. This is because all children fitting the case definition would require treatment as per the treatment guidelines. Therefore presence of any inequalities in treatment uptake would be equated to inequity.

In order to assess the effect of iCCM on appropriate treatment for pneumonia and diarrhoea, logistic regression models followed by PSM models were applied to the 2012 survey data for health seeking behaviour. The logistic regression models were used to estimate the marginal impact of exposure to iCCM on appropriate treatment for pneumonia and diarrhea while controlling for observed covariates. Despite their ability to account for possible confounders, logistic regression models are prone to the risk of selection bias. Since children exposed to iCCM may differ from those exposed elsewhere in several ways that can also influence uptake of appropriate treatment. PSM models were therefore constructed to eliminate the risk of selection bias. The propensity score generated by PSM methods is a balancing score that is believed to imitate a randomised controlled trial by generating a control and treatment group known as the ‘treated’ and ‘control’ respectively. This is because systematic differences in the characteristics of individuals who choose to participate or not to participate in a programme are removed [25, 26]. Rosenbaum (1983) showed that if one conditions on the probability that a person participates in a programme (which is iCCM in this case), based on a set observable characteristics (X) that influence programme participation (P), known as the “propensity score (Pr(X) = Probability (P =1| X))”, this person’s participation decision (P) is also independent of the potential outcomes E(Y1 and Y0) where Y1 is the expected outcome in the presence of the intervention and Y0 is the expected outcome in the absence of the intervention [25, 26]. Propensity scores are predicted probabilities, which have a continuous range known as “support” between 0 and 1 and thus the matching is done based on some intervals of this “support”, such as (0,0.1), (0.1,0.2) for individuals whose propensity score lies within the “common support”. The average treatment effects on the treated (ATT) is the mean of individual differences between the outcome of individual participants and their matched pairs and are denoted as E[Y1 – Y0| X, P = 1].

In this paper, the impact evaluation section refers to children who sought for care from a CHW for diarrhoea or pneumonia (used iCCM) as the ‘treated group’ and those who sought care from elsewhere as the ‘control group’. Therefore the term average treatment effects on the treated (ATT) has been used to refer to the effect of iCCM on appropriate treatment for pneumonia or diarrhoea.

The covariates (observable variables) included in the final PSM models included child’s age, sex of the respondent, education level of primary caretaker, socioeconomic status, concurrent infection with fever or diarrhoea, living in an peri-urban area, having no mode of transport at home, knowledge that CHWs have medicines, knowledge of danger signs for pneumonia, number of previous visits to CHWs in the past 3 months, history of hospitalisation in the last three months, and duration of illness. Logit regression was used to predict the propensity score and ATT for participation in iCCM were computed using different PSM techniques including a) kernel matching with bootstrap standard errors, STATA command attk b) stratification on the propensity score matching, STATA command atts c) nearest neighbour matching, STATA command attnd and d) radius matching of propensity scores within a calliper of 0.01, STATA command attr. Further more, sensitivity analysis using Rosenbaum bounds (mhbounds command in STATA) was conducted on the consistently significant ATT results. The purpose of the sensitivity analysis was to examine the robustness of the ATT results to unobserved confounding variables. In all of the analysis, sample weights were applied and cluster robust standard errors are reported. All computations were done in STATA 12 (College Station, TX).

## Results

Overall, there was a significant increase in the proportion of children treated appropriately for both pneumonia and diarrhoea between 2009 and 2012 (Table 1). Pneumonia cases treated with appropriate antibiotics increased significantly from 55.9 % to 66.4 % (p = 0.005) and the proportion of children receiving ORS for diarrhoea increased significantly from 34.5 % to 51.1 % (p = 0.001). The proportion receiving both ORS and zinc increased from 3.0 % to 11.8 % (p = 0.001). Table 2 shows the distribution of the indirectly standardised mean of illness prevalence and appropriate treatment across the wealth quintiles.

**Table 1:** Key indicators for disease prevalence and treatment for pneumonia and diarrhoea

Disease/treatment	2009		2012		Increase year on year percentage points	*p*-value
	N (1178)	%	N (1476)	%		
Two week prevalence of ‘fast-breathing’ pneumonia	263	22.3 %	453	30.7 %	8.4	0.000^a^
Two week prevalence of diarrhoea	168	14.6 %	229	15.6 %	1.0	0.470
‘Fast-breathing’ pneumonia treated with appropriate antibiotics	147	55.9 %	301	66.4 %	10.5	0.000^a^
Diarrhoea treated with ORS	58	34.5 %	117	51.1 %	16.6	0.001^a^
Diarrhoea treated with ORS plus zinc	5	3.0 %	27	11.8 %	8.8	0.001^a^
Treatment	First source of care 2012					*p*-value
			iCCM n (%)		Other n (%)	
‘Fast-breathing’ pneumonia treated with appropriate antibiotics (N = 453)			184 (85.6)		117 (49.2)	0.000^a^
Diarrhoea treated with ORS (N = 229)			65 (80.2)		52 (35.14)	0.000^a^
Diarrhoea treated with ORS plus zinc (N = 229)			22 (14.9)		5 (6.2)	0.056

^a^Significant at 95 % level of significant

**Table 2:** Distribution of wealth and indirectly standardized mean outcome variables for disease prevalence and treatment across wealth quintiles

Quintile	Poorest	Very poor	Poor	Less poor	Least poor
Distribution of wealth 2009	0.155	0.208	0.191	0.195	0.251
Distribution of wealth 2012	0.237	0.223	0.201	0.223	0.237
Two week prevalence of ‘fast breathing’ pneumonia in 2009	0.238	0.186	0.170	0.225	0.265
‘Fast breathing’ pneumonia treated appropriately in 2009	0.405	0.608	0.543	0.580	0. 767
Two week prevalence of ‘fast breathing’ pneumonia in 2012	0.267	0.290	0.313	0.337	0.365
‘Fast breathing’ pneumonia treated appropriately in 2012	0.406	0.531	0.755	0.595	0.523
Two week prevalence of diarrhoea in 2009	0.193	0.119	0.118	0.246	0.194
Diarrhoea treated appropriately in 2009	0.305	0.332	0.178	0.395	0.529
Two week prevalence of diarrhoea in 2012	0.094	0.168	0.152	0.166	0.102
Diarrhoea treated appropriately in 2012	0.414	0.389	0.528	0.576	0.618
Children using iCCM for ‘fast breathing’ pneumonia (2012)	0.400	0.503	0.359	0.426	0.297
Children using iCCM for diarrhoea (2012)	0.327	0.270	0.159	0.374	0.182

While reported pneumonia prevalence in 2009 was not significantly different among the poorest and least poor (CCI = 0.027; SE = 0.033), the prevalence in 2012 was higher among the least poor groups (CCI = 0.077; SE = 0.033) (Table 3). The proportion of children receiving appropriate treatment for pneumonia in 2009 was also more prevalent among the least poor (CCI = 0.152; SE = 0.092). However the proportion of children receiving appropriate antibiotics was not significantly different among the least poor and poorest in 2012 (CCI = 0.070; SE = 0.083). Diarrhoea prevalence was more concentrated among the least poor in 2009 (CCI = 0.044; SE = 0.039) but not in 2012 (CCI = -0.007; SE = 0.033). Additionally, receiving ORS for diarrhoea was more prevalent among the least poor in 2009 (CCI = 0.151; SE = 0.148) and was even increasingly more prevalent among the least poor in 2012 (CCI = 0.199; SE = 0.118). Zinc uptake in 2012 remained too low (11.8 %; 27/229) to provide a sample size large enough to warrant calculation of a concentration index.

**Table 3:** Erreygers’ corrected concentration indices (CCI) for disease prevalence, treatment uptake and horizontal inequity index (I) for the use of integrated community case management

Variable	CCI 2009	Standard error	(I)	CCI 2012	Standard error	(I)
‘Fast breathing’ pneumonia prevalence	0.027	0.033		0.077^a^	0.033	n/a
Overall antibiotic treatment	0.152	0.092	n/a	−0.070	0.083	n/a
Use of iCCM for ‘fast breathing’ pneumonia	n/a	n/a	n/a	−0.099^a^	0.073	−0.099
Diarrhoea prevalence	0.044^a^	0.039		−0.007	0.026	n/a
Overall ORS treatment	0.151^a^	0.148	0.151	0.199^a^	0.118	n/a
Use of iCCM for diarrhoea	n/a	n/a	n/a	−0.073	0.085	−0.012
ORS & zinc^b^	n/a	n/a	n/a	n/a	n/a	n/a

^a^Significant value within 95 % confidence intervals

^b^Excluded because of insufficient sample size

There was no evidence of socioeconomic inequalities in use of iCCM for children with reported fast-breathing pneumonia (CCI = -0.099; SE = 0.073) . The use of iCCM for children with supposed pneumonia seems to favour the least poor with total horizontal inequity index of -0.099. There was also no evidence of socioeconomic inequalities in use of iCCM for children with reported symptoms of diarrhoea (CCI = -0.073; SE = 0.085).

In the logistic regression models from which the marginal effects were calculated, children seeing CHWs as the first source of care were (42.2 % points, p < 0.0001) more likely to receive appropriate antibiotics for pneumonia compared to those who did not see a CHW. They were also (39.6 % points, p < 0.0001) more likely to ORS for diarrhoea compared to those who did not see a CHW. There was however no difference in the proportion of children receiving ORS-zinc combination between the treatment and control groups (p = 0.340).

**Fig. 2:**
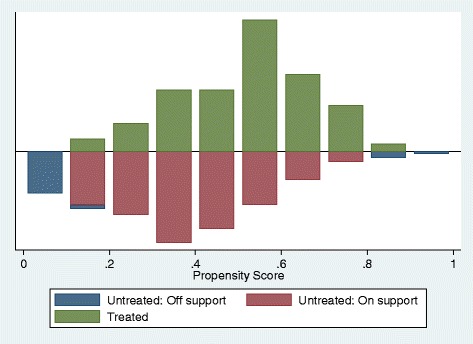
Distribution of propensity scores among the treated (iCCM) group and control group for uptake of appropriate treatment for pneumonia

**Fig. 3:**
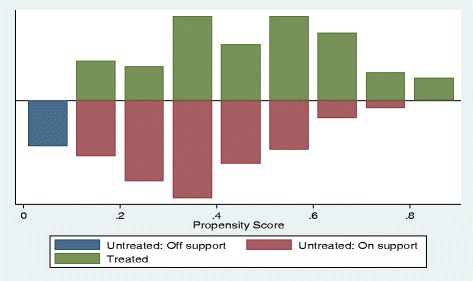
Distribution of propensity scores among the treated (iCCM) group and control group for uptake of appropriate treatment for diarrhoea

Propensity scores for 453 children with reported symptoms of pneumonia ranged from 0.11 to 0.83 with a mean (SD) of 0.45 (0.17) (Fig. 2), and from 0.10 to 0.88 with a mean (SD) of 0.41 (0.18) for 229 children with diarrhoea (Fig. 3). Each estimator generated five subgroups in which all covariates were balanced. The resulting matched treatment and control groups did not differ significantly on most covariates (Tables 4 and 5). On average, at a 5 % significance level, using the Kernel matching method, more children in the treatment (iCCM) group received appropriate antibiotics for pneumonia (ATT = 32.7 %, p < 0.001) and ORS for diarrhoea (ATT = 41.2 %, p < 0.001) compared to the control group (Table 6). No such increase was observed for children receiving ORS-zinc combination (ATT = -0.14.5 %, p <0.05). These significant iCCM programme effects were consistent across all matching estimators for both the treatment of pneumonia with appropriate antibiotics and treatment of diarrhoea with ORS but not for diarrhoea treatment with ORS-zinc combination (Table 6). The sensitivity analysis of the Q_mh + statistic, which adjusts the Mantel-Haenszel statistic downward for positive unobserved selection bias and the Q_mh- statistic, which adjusts the Mantel-Haenszel statistic downward for negative unobserved selection bias, are summarised in Table 7. The analysis shows that the ATT for appropriate treatment for pneumonia would be sensitive to bias that will increase the odds of exposure to iCCM to five. The results for appropriate treatment for diarrhoea with ORS would be sensitive to bias that would quadruple the odds of exposure to iCCM.

**Table 4:** Summary of matched and unmatched group characteristics before and after PSM for pneumonia

Variable	Unmatched and untreated	Unmatched and treated	P > |t|	Matched and untreated	Matched and treated	P > |t|
Age child	2.37	2.28	0.484	2.22	2.15	0.601
Sex caretaker	1.63	1.70	0.105	1.69	1.69	0.906
Children per household	2.00	2.02	0.824	2.06	2.04	0.82
Mother’s education	0.16	0.10	0.089	0.11	0.11	0.862
Household wealth	−0.08	−0.44	0.02	−0.46	−0.40	0.698
Fever present	0.42	0.49	0.101	0.57	0.51	0.323
Diarrhoea present	0.20	0.23	0.356	0.28	0.25	0.62
Peri-urban location	2.75	2.83	0.145	2.85	2.87	0.793
No transport available at home	0.54	0.44	0.032	0.46	0.43	0.66
Aware that CHWs have drugs	1.74	1.34	0.018	1.41	1.25	0.237
Aware of pneumonia danger signs	0.12	0.16	0.151	0.13	0.16	0.441
Previous visits to CHWs	1.57	2.17	0.001	2.43	2.17	0.224
Number of previous hospitalisation	6.59	4.55	0.344	0.96	1.32	0.727
Duration of illness	6.89	5.61	0.071	5.68	5.56	0.772

N.B. The term treated in the table refers to children exposed to the iCCM programme

**Table 5:** Summary of matched and unmatched group characteristics before and after PSM for diarrhoea

Variable	Unmatched and untreated	Unmatched and treated	P > |t|	Matched and untreated	Matched and treated	P > |t|
Age child	1.92	1.87	0.727	1.67	1.81	0.436
Sex caretaker	1.63	1.57	0.368	1.58	1.54	0.622
Children per household	2.01	2.04	0.856	1.92	2.09	0.216
Mother’s education	0.11	0.10	0.815	0.20	0.11	0.114
Household wealth	−0.41	−0.68	0.147	−0.48	−0.69	0.32
Fever present	0.45	0.60	0.028	0.61	0.58	0.74
Cough + fast breathing present	0.38	0.49	0.091	0.46	0.47	0.87
Peri-urban location	2.76	2.78	0.783	2.51	2.81	0.005
No transport available at home	0.44	0.68	0.001	0.59	0.66	0.398
Aware that CHWs have drugs	1.59	1.26	0.11	1.44	1.20	0.262
Previous visits to CHW	1.89	2.32	0.122	2.69	2.34	0.265
Number of previous hospitalisation	6.24	1.41	0.084	0.28	0.20	0.542
Duration of illness	6.49	6.54	0.974	7.30	6.61	0.306

N.B. The term treated in the table refers to children exposed to the iCCM programme

**Table 6:** Average treatment effects on the treated (ATT) for appropriate treatment of diarrhoea and pneumonia

Matching method	Outcome	Number in ‘treated’	Number in ‘control’	ATT	Standard error	*t*-test	*p*-value
Kernel	Received ORS for diarrhoea	74	111	0.412	0.076	5.398	< 0.001^a^
	Received ORS & zinc for diarrhoea	74	111	−0.146	0.069	−2.112	< 0.05^a^
Nearest neighbour	Received ORS for diarrhoea	74	44	0.419	0.110	3.820	< 0.001^a^
	Received ORS & zinc for diarrhoea	74	44	−0.095	0.078	−1.042	> 0.05
Stratification	Received ORS for diarrhoea	70	115	0.412	0.076	5.398	< 0.001^a^
	Received ORS & zinc for diarrhoea	70	115	−0.130	0.058	−2.236	< 0.05^a^
Radius	Received ORS for diarrhoea	63	89	0.442	0.076	5.812	< 0.001^a^
	Received ORS & zinc for diarrhoea	63	89	−0.099	0.056	−1.883	> 0.05
Kernel	Antibiotic for pneumonia	166	190	0.327	0.050	6.548	< 0.001^a^
Nearest neighbour	Antibiotic for pneumonia	166	82	0.289	0.072	4.024	< 0.001^a^
Stratification	Antibiotic for pneumonia	163	193	0.331	0.053	6.229	< 0.001^a^
Radius	Antibiotic for pneumonia	156	177	0.328	0.052	6.273	< 0.001^a^

^a^Significant value within 95 % confidence intervals

**Table 7:** A sensitivity analysis using Mantel-Haenszel bounds for receiving an appropriate antibiotic for pneumonia or ORS for diarrhoea

Outcome	Gamma (C)	Q_mh+	Q_mh2	p_mh+	p_mh2
Antibiotic for pneumonia	1	7.231	7.231	< 0.0001	< 0.0001
	2	4.344	10.442	< 0.0001	0.000
	3	2.754	12.501	0.003	0.000
	4	1.659	14.063	0.048	0.000
	5	0.822	15.343	0.205	0.000
ORS for diarrhoea	1	5.528	5.528	< 0.0001	< 0.0001
	2	3.474	7.821	0.000	< 0.0001
	3	2.336	9.278	0.010	0.000
	4	1.552	10.380	0.060	0.000
	5	0.953	11.280	0.170	0.000

Gamma: odds of differential assignment due to unobserved factors

Q_mh+: Mantel-Haenszel statistic (assumption: overestimation of treatment effect)

Q_mh2: Mantel-Haenszel statistic (assumption: underestimation of treatment effect)

p_mh+: significance level (assumption: overestimation of treatment effect)

p_mh2: significance level (assumption: underestimation of treatment effect)

## Discussion

Results from this study point to overall improvement in uptake of appropriate treatment for pneumonia and diarrhoea across all socioeconomic strata following introduction of iCCM. There were also no significant inequalities in use of iCCM observed despite the common observation that introduction of new interventions may worsen inequities in the short run [14, 27]. The occurrence of inequities in access to treatment within rural communities that are assumed to be uniformly poor has previously been ascertained [28]. However, in this study no discrepancies were observed among children from different socioeconomic groups who were eligible for treatment and who received it. This might be due to presence of under and over reporting of ill health by poorer and less poor groups, respectively, leading to small or no socioeconomic inequalities being observed [24]. While this is a potential bias it is unlikely to be the case, given the nature of free treatment offered in iCCM.

The use of ORS was more concentrated among the least poor in 2009 who also seemed to carry more of the diarrhoea burden at that time. However, as time progressed, the least poor groups were disproportionately receiving more of the ORS for diarrhoea despite no obvious inequalities in the prevalence of diarrhoea among the groups. This could be due to the fact that more of the poorest children with diarrhoea were seeking for health care from outside the iCCM intervention and were therefore unable to access ORS. This can be seen in Fig. 1, which shows that more children (81 versus 148) with diarrhoea did not seek for health care from CHWs.

There is a significant increase in the total number of children classified, as pneumonia in 2012 however there was no significant difference in the proportion of children seeking for care from iCCM and elsewhere. This finding is comparable to what has been observed in Sierra-Leone [29] and could be due to the fact that introduction of the iCCM programme raised awareness among community members who were then more likely to report cough and fast-breathing during an illness episode upon seeking treatment from outside the home.

There were highly significant iCCM programme effects observed for uptake of both appropriate pneumonia and diarrhoea treatment. More of the children with reported pneumonia symptoms in the treatment group received an appropriate antibiotic compared to the children in the control group. There were also significantly more children receiving ORS for diarrhoea in the treatment group compared to the control group. However, zinc uptake remained low and did not differ significantly between the treatment and control groups. Although some authors have argued that there is insufficient evidence on efficacy and effectiveness of community management of pneumonia [12], the data suggest that if the WHO-iCCM algorithm [30] could be followed by CHWs, the implementation of iCCM alone in this predominantly rural setting would lead to 32.7 % increase in the number of children with pneumonia symptoms receiving appropriate antibiotics, and a 40.0 % increase in the number of children with diarrhoea receiving ORS. The high ORS uptake combined with low zinc uptake highlights the need to explore the untapped strategy of co-packaging ORS and zinc, which has proven to improve uptake of diarrhoea treatment elsewhere [31].

Study limitations and methodological considerations include reporting bias of symptoms by caregivers, leading to pneumonia misclassification. Also, PSM is known to be prone to bias arising from endogenous covariates and model misspecification [25, 26]. However the use of PSM in this analysis is justifiable as there was no CHW/iCCM intervention (treatment group) at baseline. PSM is known to be superior to traditional logistic regression models, which account for many possible confounders but for which the risk of selection bias often remains [26]. Efforts were made to include all necessary variables affecting programme selection available in the dataset to mitigate bias. A sensitivity analysis was also done to see how the results would be affected by varying levels of bias. Although sample size calculations were based on a representative sample, there were inevitable limitations of using household survey data for equity analysis [32]. For example there were small samples sizes in some sub-populations such as the proportion of children receiving zinc for diarrhoea, which prevented further meaningful analysis.

## Conclusion

In view of limited human resources for health, large scale implementation of CHWs delivering integrated child health services could lead to significant gains in the proportion of sick children receiving appropriate treatment. Yet careful consideration of the common challenges CHWs face delivering high quality care in an isolated rural environment is needed, especially to address lack of supervision and drug supply, which negatively affect CHW performance and subsequently programme outputs [12, 33–35]. There is also a need to implement and evaluate simple interventions that can increase uptake of zinc, like co-packaging of ORS with zinc, as these might serve to improve overall uptake of effective diarrhoeal treatments [31].

## Abbreviations

ATT: Average treatment effects on the treated
CHW: Community health worker
CI: Confidence interval
CCI: Corrected concentration index
CCM: Community case management
iCCM: Integrated community case management
OR: Odds ratio
ORS: Oral rehydration salts
PSM: Propensity score matching
SE: Standard error
VHT: Village health team
UBOS: Uganda bureau of statistics
UNICEF: United Nations Children’s Fund
